# Effect of preoperative coronary CT for planning of percutaneous coronary intervention for complex chronic total occlusion (CTS-C-CTOPCI): study protocol for an open-label randomised controlled trial

**DOI:** 10.1186/s13063-023-07458-y

**Published:** 2023-08-29

**Authors:** Eugenio La Scala, Jean-Pascal Peyre, Eric Maupas, ReSurg, Jacobus H. Muller, Mo Saffarini, CT-CTO PCI Study Group, Alfredo Galassi, Giuseppe Vadalà, Luca Grancini, Daniele Andreini, Antoine Boge, Jerome Brunet

**Affiliations:** 1 ELSAN Group, Polyclinique Les Fleurs, 332 avenue Frédéric Mistral, Ollioules, 83190 France; 2 ELSAN Group, Clinique Rhône Durance, 1750 chemin du Lavarin, Avignon, 84000 France; 3 ELSAN Group, Hôpital Privé Les Franciscaines, 3 Rue Jean Bouin, Nîmes, 30000 France

**Keywords:** Chronic total occlusion, Percutaneous coronary intervention, Coronary computed tomographic angiography

## Abstract

**Background:**

Treatment of chronic total occlusion (CTO) by percutaneous coronary intervention (PCI) is associated with the difficulty of guidewire manipulation through the occluded segment, particularly when there is hard tissue due to calcification. The purpose of this randomised controlled trial is to determine whether improved planning of CTO-PCI using coronary computed tomographic angiography (CCTA) (versus conventional angiography) increases success rates of wire crossing in ≤ 60 min in difficult cases.

**Methods:**

This is a randomised controlled open-label multi-centre trial in a superiority framework with 1:1 allocation ratio. Participants (*n* = 130) will be randomised into two groups: the study group who will receive standard of care with the addition of preoperative coronary computed tomographic angiography (CT group), and the control group that will receive standard of care (angiography group). The primary endpoint will be the rate of successful wire crossing in ≤ 60 min in complex CTO (J-CTO ≥ 2). Wire crossing will be considered successful if TIMI flow 3 is restored and residual stenosis is < 30%. The safety endpoint will be mortality due to the intervention or major adverse cardiac events (MACE). Secondary endpoints are success rates at any time; total time of PCI; time of wire crossing; rate of PCI complications; radiation levels during PCI; volume of iodine contrast medium administered; and cost of the PCI.

**Discussion:**

This randomised trial will provide insight into whether pre-procedural CCTA as opposed to conventional angiography for planning of CTO-PCI yield higher success rates of wire crossing in ≤ 60 min. Potential benefits of CCTA include shorter successful procedure times of CTO-PCI leading to less irradiation and contrast medium with lower complication rates.

**Trial registration:**

Clinical Trials.gov NCT04549896. Registered on December 21, 2021.

## Administrative information

Note: the numbers in curly brackets in this protocol refer to SPIRIT checklist item numbers. The order of the items has been modified to group similar items (see http://www.equator-network.org/reporting-guidelines/spirit-2013-statement-defining-standard-protocol-items-for-clinical-trials/).

**Table:** 

Title {1}	Effect of preoperative coronary CT for planning of percutaneous coronary intervention for complex chronic total occlusion (CTS-C- CTOPCI): study protocol for a randomised controlled trial
Trial registration {2a and 2b}.	Clinical Trials.gov NCT04549896, December 21, 2021
Protocol version {3}	ClinicalTrials.gov ID: NCT04549896; Unique Protocol ID: CTS-C-CTOPCI
Funding {4}	***ELSAN Comité Orientation Scientifique***
Author details {5a}	Dr Eugenio La Scala,^1^ MD Dr Jean-Pascal Peyre,^2^ MD Dr Eric Maupas,^3^ MD ReSurg,^4^ CT-CTO PCI Study Group,^5^
	1. ELSAN Group, Polyclinique Les Fleurs, 332 avenue Frédéric Mistral, 83190, Ollioules, France 2. ELSAN Group, Clinique Rhône Durance 1750 chemin du Lavarin, 84000 Avignon, France 3. ELSAN Group, Hôpital Privé Les Franciscaines, 3 Rue Jean Bouin, 30000 Nîmes, France 4. ReSurg: Jacobus H. Muller, PhD, ReSurg SA, Switzerland. / Mo Saffarini, M.Eng, MBA, ReSurg SA, Switzerland 5. CT-CTO PCI Study Group: Prof. Alfredo Galassi, PhD, Chair of Cardiovascular Medicine, Department of PROMISE, University of Palermo, Italy. / 1. Dr Giuseppe Vadalà, MD, University Hospital P.Giaccone, Palermo, Italy. 2. Prof. Alfredo Galassi, PhD, Chair of Cardiovascular Medicine, Department of PROMISE, University of Palermo, Italy. 3. / Dr Luca Grancini, MD, IRCCS Ospedale Galeazzi S.Ambrogio, via Cristina di Belgioioso 173, 20157, Milan, Italy. / Prof. Daniele Andreini, IRCCS Ospedale Galeazzi S.Ambrogio, via Cristina di Belgioioso 173, 20157, Milan, Italy. / Dr. Antoine Boge, ELSAN Group, Clinique Rhône Durance 1750 chemin du Lavarin, 84000 Avignon, France. / Dr. Jerome Brunet, ELSAN Group, Clinique Rhône Durance 1750 chemin du Lavarin, 84000 Avignon, France.
Name and contact information for the trial sponsor {5b}	Polyclinique Les Fleurs ELSAN Polyclinique Les Fleurs, 332 avenue Frédéric Mistral, 83190 Ollioules, France
Role of sponsor {5c}	Finance of services inherent to the research (methodology, ethical and regulatory submission, database, and medical writing).

## Introduction

### Background and rationale {6a}

Chronic total occlusion (CTO) refers to the complete obstruction of a coronary artery classified as Thrombolysis in Myocardial Infarction (TIMI) 0 flow for at least 3 months and is frequently diagnosed in patients with coronary artery disease [1–3]. Treatment of CTO can be categorised as non-invasive medical (aspirin, anti-cholesterol drugs, β-blockers, ACE inhibitors, nitrates, etc.), minimally invasive interventional (percutaneous coronary intervention (PCI)), or invasive surgical (bypass surgery). Since medical approaches often do not reduce symptoms or improve quality of life, minimally invasive interventions should be considered, especially in cases with angor/dyspnea, ischemia or ejection fraction reduction associated with viability of the myocardial wall, originally perfused by the occluded segment. The main challenge of CTO-PCI is the difficulty of guidewire manipulation in the correct trajectory through the occluded segment, particularly when there is hard tissue due to calcification. Technical developments and advances in algorithmic approaches to CTO-PCI resulted in higher success rates, reduced complications and improved procedural efficiency [3–5]; however, procedural success rates of CTO-PCI across studies range from 61 to 99% and are associated with procedural and anatomical difficulties [2]. The cardiologist has the choice of different PCI strategies (anterograde, retrograde, true to true, dissection re-entry) using most of the time a double vascular access (double radial, radial-femoral or double femoral). Due to the limited information about the occlusion anatomy, the antegrade wire escalation technique is often the first step to test the occlusion with softer wires before going up with stiffness and weight.

Different clinical models for lesion assessment have been proposed to assist cardiologists in choosing the ideal approach [2], of which the 5-point Japanese Multicentre CTO Registry (J-CTO) score is the most common [6]. The J-CTO score predicts the complexity of CTO-PCI as easy (0), intermediate (1), difficult (2), or very difficult (≥3), by assessing four characteristic features of the occluded section by conventional angiography: proximal cap shape, presence of calcifications, degree of bending, length of occlusion, and whether CTO-PCI has been attempted before. Nonetheless, scores derived from conventional angiography may not be sufficient to predict CTO-PCI difficulty, duration and outcome [7]. Coronary computed tomographic angiography (CCTA) enables non-invasive visualisation of proximal cap position and characteristics, of exact vessel trajectory and calcium distribution. This kind of information is less precise using conventional angiography. CCTA therefore offers a better evaluation of complex CTO, which could improve planning and outcomes of CTO-PCI, shorter procedure times, less radiation and less iodine contrast injection, as well as reduced costs and perhaps a higher success rate.

The purpose of this randomised controlled trial is to determine whether improved planning of CTO-PCI using CCTA (versus conventional angiography) increases success rates of wire crossing in ≤60 min in difficult cases (J-CTO ≥2). The primary endpoint will be the proportion of successful procedures, defined by TIMI flow 3 and stenosis < 30%, with wire crossing completed in ≤60 min. Secondary endpoints will include the proportions of successful procedures completed regardless the timeframe, time of wire crossing, radiation levels and volume of iodine contrast injected, and procedural complications. Safety endpoints will be mortality due to the procedure or any major adverse cardiac events (MACE).

### Objectives {7}

To determine whether improved planning of CTO-PCI using CCTA (versus conventional angiography) increases success rates (TIMI flow 3 and stenosis < 30%) of wire crossing in ≤60 min in difficult cases (J-CTO ≥2). To compare the proportions of successful procedures completed regardless the timeframe, time of wire crossing, radiation levels and volume of iodine contrast injected, procedural complications, and any major adverse cardiac events (MACE).

### Trial design {8}

A randomised controlled open-label multi-centre trial in a superiority framework with 1:1 allocation ratio.

## Methods: participants, interventions and outcomes

### Study setting {9}

The study setting is based at five centres:

- Polyclinique Les Fleurs, ELSAN Group, 332 avenue Fréderic Mistral, 83190 Ollioules, France
- Clinique Rhône Durance, ELSAN Group, 1750 chemin du Lavarin, 84000 Avignon, France
- Hôpital Privé Les Franciscaines, ELSAN Group, 3 rue Jean Bouin 30032 Nîmes, France
- Aziende Ospedaliera Universitaria Paolo Giaccone, via Del Vespro 129, 90127 Palermo, Italy
- IRCCS Ospedale Galeazzi S.Ambrogio, via Cristina di Belgioioso 173, 20157, Milan, Italy

### Eligibility criteria {10}

The authors will enrol 130 patients undergoing difficult CTO-PCI.

The inclusion criteria will be:

1. Men and women aged 18 to 90 years with body mass index (BMI) ≤ 40 kg/m^2^;
2. Coronary artery chronic total occlusion (TIMI flow 0 for > 3 months) associated with myocardial viability (intended like normokinetic/hypokinetic wall motion or instrumental data in favour of viability) associated with ≥ 2 Canadian Cardiovascular Society (CCS) angina or ≥ 2 New York Heart Association (NYHA) dyspnoea or documented ischemia or ejection fraction < 50%;
3. Coronary angiography J-CTO score ≥ 2;
4. Understanding of, and agreement to comply with the study procedures;
5. Signature of informed consent form prior to any study-related procedures; and
6. Affiliation to social security scheme.

The exclusion criteria will be:

1. Coronary angiography J-CTO score of 0 or 1;
2. Pregnancy or other protection by law;
3. Very frequent irregular cardiac rhythm (arrythmia?/tachycardia?) > 100 beats/min;
4. Concomitant pathologies that introduce additional risks, such as hemodynamic instability, anaemia < 9 g/dl, gastrointestinal bleeding, thrombocytopenia with < 50,000/mm^3^ platelets count or severe valvular disease; or
5. CT-scan performed before inclusion into the study.

### Who will take informed consent? {26a}

Participants in the study will sign the written informed consent which will be collected by the cardiologist (principal investigator) who will perform the CTO-PCI.

### Additional consent provisions for collection and use of participant data and biological specimens {26b}

Not applicable.

## Interventions

### Explanation for the choice of comparators {6b}

Participants will be randomised into two groups with a 1:1 allocation: the study group who will receive standard of care with the addition of preoperative CCTA-scan (CT group), and the control group that will receive standard of care (angiography group).

### Intervention description {11a}

#### Preoperative assessment

The following assessments will be performed as part of the standard of care for the diagnosis of CTO:

1. Conventional coronary angiography
2. If symptoms and viability are not established through angiography, the patient will undergo one of the following:
  a Stress electrocardiogram (ECG)
  b Cardiac magnetic resonance imaging (MRI)
  c Stress echocardiography
  d Myocardial scintigraphy
3. Collection of biological markers: troponin, creatine kinase, creatinine, clearance of creatinine, haemoglobin. Preoperative angiography will be performed for all patients in ambulatory settings under local anaesthesia, with either a radial artery or femoral artery approach, with the former being the first choice if the artery is of good quality. An introducer sheath measuring 4 or 5 French (Fr.) is advanced within the artery using the Seldinger technique, and a catheter is advanced through the introducer up to the heart. A 6–8 cc iodine contrast injection in the coronary artery ostia allows visualisation of coronary artery anatomy under radiography. A picture registration is obtained and archived for planning of CTO-PCI. During the procedure heparin and nitrates are administrated.

If conventional angiography indicates a J-CTO score ≥2, the patient will be enrolled in the study, if they meet the eligibility criteria. Patients will then be randomly allocated to the CT group or angiography group. Patients in the CT group will undergo an additional CCTA scan (GE CT Revolution Scan 256; Iomeron 350 iodine contrast). The total dose length product (DLP, mGy/cm^2^) and computed tomography dose index (CTDI, mGy) will be recorded. Iodine contrast for the CCTA will be 55ml (10ml saline–55ml Iomeron 350–20ml saline at 5ml/s).

For the CT group, the PCI strategy will be based on:

1. J-CTO scores derived from conventional angiography as well as CCTA, to assess five characteristic features of the occluded section: proximal cap shape, presence of calcifications, degree of bending, and length of occlusion, as well as whether CTO-PCI has been attempted before.
2. CT RECTOR (Computed Tomography Registry of Chronic Total Occlusion Revascularization) score, to assess: the presence of multiple occlusions, the stump morphology, degree of calcification and bending, as well as whether previous CTO-PCI has been attempted before.
3. Calcium score of the occluded artery and occluded segment to assess the calcium distribution (full moon, eccentric > 50% or < 50%, circumferential) at the proximal and distal cap as well as the body of the occlusion; and
4. Inspection of the occluded coronary artery anatomy as visualised on the CCTA.

For the angiography group, the PCI strategy will be based on the J-CTO score derived from conventional angiography. The occlusion is assessed based on its proximal cap shape, presence of calcifications, degree of bending, and length. The patient history is assessed to determine if CTO-PCI has been attempted before.

### CTO-PCI

The recanalisation procedure is the most complex type of percutaneous coronary angioplasty. A double radial access or a radial/femoral access is used in most of the patients. A simple arterial access is used in the case of ipsilateral collateral circulation. The first and most complex step is to go through the occluded segment with a 0.08- to 0.014-inch tip guidewire to access the true lumen in the distal part of the artery, by one or more of the following approaches: anterograde, retrograde, dissection/re-entry (anterograde or retrograde). A double arterial access is often necessary to visualise the distal part of the artery through the collateral circulation and to go retrograde if necessary.

### Criteria for discontinuing or modifying allocated interventions {11b}

Treatment for some individuals that fulfilled the eligibility criteria and were allocated into the study or control group may be discontinued or modified if they present with:

1. New disease that requires an urgent intervention which doesn’t permit antiplatelet treatment due to bleeding risk,
2. COVID-19 infection,
3. Septic status, or
4. A disease that affects the patient’s autonomy.

### Strategies to improve adherence to interventions {11c}

Not applicable, as the nature of the intervention is a one-off delivery of CTO PCI, rather than a series of steps that require adherence.

### Relevant concomitant care permitted or prohibited during the trial {11d}

As per standard of care, mandatory dual antiplatelet therapy has to be taken in both the CT group and angiography group due to PCI, which is composed of one of three options:

1. Option 1: Aspirin 150 mg/day and clopidogrel 150 mg/day for the first month; aspirin 150 mg/day and clopidogrel 75 mg/day for the 5 months following; and only aspirin 160 mg/day without interruption after 6 months. Load of clopidogrel: 600 mg once, more than 12 h before the PCI.
2. Option 2: Aspirin 75 mg/day and ticagrelor 90 mg 2 times/day for the first 6 months; aspirin 160 mg/day alone without interruption after 6 months. Load of ticagrelor: 180 mg once, more than 6 h before the PCI
3. Option 3: Aspirin 75 mg/ day and prasugrel 10 mg/day for the first 6 months; aspirin 160 mg/day without interruption after 6 months. Load of prasugrel: 60 mg once, more than 6 h before the PCI

Single antiplatelet therapy will be administered in case of concomitant anticoagulant treatment, comprising clopidogrel 150mg/day for 1 month and then 75mg/day for 6 months, aspirin 75mg/day or clopidogrel 75mg day after 6 months. Load of clopidogrel: 600mg more than 12 h before the PCI.

In case of chronic renal insufficiency (clearance of creatinine <47ml/min), hydration with 1000 ml saline will be necessary before the CT scan and before the CTO-PCI. In case of allergy to contrast medium, antihistamines and corticosteroids will be taken before the CT scan and before the CTO-PCI.

The immediate result of the CTO-PCI procedure will be evaluated during the first two days of hospitalisation after the procedure. As part of standard of care, treatments and activities will be monitored:

1. Metformin will be suspended 24 h before and 48 h after CTO-PCI.
2. Anticoagulants will be suspended 72 h before CTO-PCI.
3. Blood samples will be collected before and one day after CTO-PCI to evaluate coagulation, renal function, troponin, creatine kinase, platelet count and haematocrit.

### Provisions for post-trial care {30}

There is no anticipated harm and compensation for trial participation. Patients will undergo routine follow-ups with their cardiologist, and participation in the study will stop at 6 months after CTO-PCI.

### Outcomes {12}

The primary objective of this study is to compare rates of successful wire crossing in ≤60 min in complex CTO (J-CTO ≥2) among the CT group and angiography group. Wire crossing will be considered successful if TIMI flow 3 is restored and residual stenosis is <30%. TIMI flow and residual stenosis will be evaluated by a pool of two cardiologists who are not directly involved in the procedure, and who are blinded to group allocation. The TIMI flow scale is classified as Grade 0 to 3 (Table 1).

**Table 1:** TIMI flow grades

Grade 0	No flow
Grade 1	penetration without perfusion in the distal part of the artery
Grade 2	partial perfusion, the iodine contrast goes across the obstruction and fills the distal part of the artery, but the flow and the clearance of the iodine contrast are perceptibly slower if compared to the other non-occluded segments/arteries
Grade 3	complete perfusion, anterograde flow into the distal part of the artery with rapid filling and clearance if compared to the other segments/arteries.

The secondary objectives of the study are to compare:

1. Success rates at any time
2. Total time of PCI (minutes)
3. Time of wire crossing
4. Rate of PCI complications (Major Adverse Cardiac Events, MACE): death, coronary perforation with cardiac tamponade requiring pericardiocentesis, myocardial infarction (if elevation of troponin or creatine kinase associated with change in electrocardiogram), stroke, major bleeding with loss of haemoglobin > 3 g/dl.
5. Radiation levels during PCI expressed as transferred kinetic energy (air Kerma, Gy), dose area product (DAP, mGycm^2^), and fluoroscopy time (minutes).
6. Volume of iodine contrast medium administered during PCI (millilitres).
7. Cost of the PCI (euros) CTTA (CT group) Angiography Preoperative assessment, as summarised in the material invoice of every procedure.

Renal adverse events will be assessed after diagnostic coronary angiography, CCTA (CT group only), and CTO-PCI. Clinically relevant findings (like contrast allergic reaction) will be reported as adverse events. Their relationship with the CT scan or CTO-PCI will be reported. Other symptoms, such as angina CCS class, dyspnea NYHA class, MACE and renal failure will be recorded. Biologic markers, such as troponin, creatine kinase, creatinine, clearance of creatinine, and haemoglobin, will be recorded after CTO-PCI.

### Participant timeline {13}

The period of enrolment will span a 24-month period, whereas patient participation will span a 6- to 12-month period that comprise of 4 visits for the CT-scan groups and 3 visits for the angiography group (Table 2).

**Table 2:** Participant timeline

Timepoint	Enrolment	Allocation	CT-scan	CTO-PCI	Immediate follow-up	Close-out
	D0 −30 to 180 days ± 10 days	D0 −20 to 90 days ± 10 days	D0 −1 to 30 days	D0	D0 +2 to 4 days	D0 +180 days
Enrolment:	X					
Eligibility screen	X					
Informed consent	X					
Allocation		X				
CT scan			X			
Interventions:						
Comparator (wCT)			X	X		
Control (w/oCT)				X		
Assessment						
Demographic data	X					
Clinical data	X					
Risk factors	X					
Electrocardiogram	X					
Viability/ischemia test	X					
Coronary anatomy, EF and wall motion (echocardiogram or angiography)	X					
J-CTO scoring (angiography)	X					
Blood sampling	X				X	
ACT complications					X	
Adverse events	X				X	X
Symptoms, renal failure	X				X	X
MACE					X	X
TIMI flow	X				X	X

### Sample size {14}

A large registry-based study [4] on angiography-based CTO PCI revealed a procedural success rate of 87% (*n* = 3122). Two comparative studies [8, 9] on angiography-based CTO PCI versus CCTA-based CTO PCI reported success rates of respectively 90% (*n* = 30) vs. 63% (*n* = 43) and 93.5% (*n* = 187) vs. 84% (*n* = 168). Therefore, assuming a 90% success rate after 60 minutes wire manipulation in the CCTA-based CTO PCI group and 70% success rate after 60 minutes wire manipulation in the angiography-based CTO PCI group, an a priori sample size calculation revealed a minimum of 56 patients per group to detect a statistically significant difference of 20% with a power of 80% (*α* = 0.05). Assuming that <15% of patients will withdraw from the study, cross-over between groups, be lost to follow-up or have incomplete records, 65 patients will be included per group, amounting to a total of 130 patients to be included in the study.

### Recruitment {15}

Patients compliant with inclusion and exclusion criteria will be invited to participate in the study. After patients are presented with the informed consent form, they will be given sufficient time to decide if they want to take part in the study.

## Assignment of interventions: allocation

### Sequence generation {16a}

An unstratified randomisation sequence will be generated before the commencement of the study by a software algorithm (IWRS, ClinInfo SAS, Lyon, France) and to be used in assigning patients to the CT group (A) or angiography group (B) in a 1:1 ratio. The randomisation list will consist of fixed block sizes of 4, e.g. AABB–BAAB–BABA–ABBA.

### Concealment mechanism {16b}

The randomisation list generated in the IWRS software will be concealed from staff as well as patients. Only once the patient is included in the trial, will the treatment arm be assigned to the patient by the software, and reflected in the patient’s electronic case report form (eCRF) which is only accessible by the study staff. Patients will be blinded to their allocated group up to the point that they receive the treatment—patients will be aware of whether they receive CCTA before the CTO PCI.

### Implementation {16c}

Upon enrolment, the IWRS software will automatically assign the patient to a treatment arm—CT group (A) or angiography group (B)—without any input from the study staff. As patients are enrolled in the study the next number in the randomisation list will be assigned to the patient and indicated in their eCRF.

## Assignment of interventions: blinding

### Who will be blinded {17a}

This is an open-label study for the patient and treating cardiologist, but blinded for two cardiologists who will evaluate TIMI flow and residual stenosis after CTO-PCI.

### Procedure for unblinding if needed {17b}

Not applicable, as this is an open-label study.

## Data collection and management

### Plans for assessment and collection of outcomes {18a}

The following datasets will be defined:

1. Intention-to-treat (effect of assigning the treatment): This dataset will include all enrolled patients allocated to either the CT group or the angiography group, irrespective of whether they undergo CTO-PCI, cross-over between treatment arms, or have incomplete post-intervention data.
2. Per Protocol (effect of receiving the treatment): This dataset will include enrolled patients allocated to either the CT group or the angiography group, who undergo CTO-PCI, remain in their allocated treatment arm, and have complete post-intervention data (TIMI flow and residual stenosis available after PCI).
3. As-treated (effect of adherence to a treatment arm): This dataset will include enrolled patients allocated to either the CT group or the angiography group, who undergo CTO-PCI, irrespective of whether they cross-over between treatment arms, and who have complete post-intervention data (TIMI flow and residual stenosis available after PCI).

The following information will be included on the eCRF after enrolment and allocation:

1. Patients’ characteristics: age (years), sex, weight (kg), height (cm), and pregnancy test.
2. Risk factors: smoking status, diabetes, hyperlipidaemia, hypertension, cardiovascular disease family history
3. Symptoms related to the pathology (angina, dyspnea)
4. Clinical/instrumental data: ECG, angiographic ejection fraction (EF), regional wall motion, ischemia, viability.
5. Laboratory data: Troponin, creatine kinase, creatinine, clearance of creatinine, haemoglobin.
6. Anamnestic data: previous PCI, previous CTO-PCI attempt without success, previous bypass surgery.
7. Relevant comorbidity (renal function disease, pulmonary disease, peripheral artery disease, others), arrhythmias, allergy
8. Coronary anatomy based on coronary angiography and to calculate the J-CTO score for all the patients. For the CT group, coronary anatomy will be reassessed with the CCTA and in addition to the J-CTO Score based on the CCTA, the CT-RECTOR and calcium scores will be calculated.

The primary endpoint, that is the success rate of difficult CTO-PCI in ≤60 min wire manipulation, will be assessed for each of the three datasets, i.e. intention-to-treat, per-protocol and as-treated, after the CTO-PCI (D0, Table 2). The TIMI flow and residual stenosis after the procedure will be recorded for each of the three datasets, i.e. intention-to-treat, per-protocol and as-treated, and will be assessed at immediate follow-up (D0 + 2 to 4 days) and final follow-up (D0 + 180 days). Laboratory parameters will be recorded for each of the three datasets, i.e. intention-to-treat, per-protocol and as-treated, and will be assessed at immediate follow-up (D0 + 2 to 4 days). The success rate of the complex CTO-PCI between both groups will be calculated for each of the three datasets, i.e. intention-to-treat, per-protocol and as-treated, at immediate follow-up CTO-PCI (D0 + 2 to 4 days) and final follow-up (D0 + 180 days). For all three datasets, i.e. Intention-to-treat, Per-protocol and As-treated, all adverse events will be recorded according to the corresponding system organ class (SOC) and preferred term (PT) using the MedDRA terminology. Creatinine levels after the CTO-PCI, summarising the renal adverse events, will be described in both groups. Symptoms (angina CCS class/dyspnea NYHA class), MACE, and renal failure will be evaluated at the CTO-PCI visit and end of study visit.

### Plans to promote participant retention and complete follow-up {18b}

The data for primary and secondary endpoints will be collected during CTO-PCI, and the risk of missing data is therefore small (5% max). If a patient withdraws prematurely from the study they will not be replaced, and reasons for patient withdrawal must be documented in the eCRF. For patients lost to follow-up, attempts to contact the patient must be made and documented in the patient’s eCRF.

### Data management {19}

Data management will be completed using good clinical practices. All data will be entered, and a manual review will be done to avoid data entry error. The data will be entered into a validated database. The main author will be responsible for data processing, in accordance with the sponsor data management procedures. Validation checks will be performed and documented to ensure accurate, consistent, and reliable data. Data identified as erroneous, or data that are missing, will be referred to the investigative site for resolution through data queries. Correction procedures will be tracked. The database will be locked once quality assurance procedures have been completed prior to statistical analysis.

For medical information, the following dictionaries will be used:

1. The latest version at the time of study starts of the Medical Dictionary for Regulatory Activities for medical history and adverse events.
2. The latest version at the time of study starts (and each subsequent update as they become available throughout the duration of the study) of the World Health Organization Drug Dictionary for prior and concomitant medications.

### Confidentiality {27}

In accordance with the legal provisions in force (Articles L.1121-3 and R5121.13 of the Public Health Code), persons having direct access to the source data will take all necessary precautions to ensure the confidentiality of information related to experimental drugs, research, persons included in the study and especially their personal identity and the research results. Those persons, as well as the investigators themselves, are subject to professional secrecy. Each enrolled patient will have a personal eCRF with a unique identifier, in which all data related to the study will be recorded. Data collection will be the responsibility of the clinical trial staff at the site under the supervision of the site investigator. The investigator is responsible for ensuring the accuracy, completeness, legibility, and timeliness of the data entry. All source documents should be completed in a neat, legible manner to ensure accurate interpretation of data. All information required for the protocol has to be entered in the eCRF, and the investigator has to provide justification in cases of missing data entries. During the biomedical research or at the end of it, the data collected on the persons who are suitable and transmitted to the main author by the investigators will be anonymised, using the unique identifier of the case report form, to hide their name, address, contact information and date of birth. The trial steering committee will ensure that each enrolled patient provided written informed consent for access and use of their anonymised data.

### Plans for collection, laboratory evaluation and storage of biological specimens for genetic or molecular analysis in this trial/future use {33}

Not applicable as no biological samples will be collected for future analysis.

## Statistical methods

### Statistical methods for primary and secondary outcomes {20a}

Continuous variables will be reported as means, standard deviations, medians, and ranges, whereas categorical variables will be reported as numbers and proportions. Missing data will be considered separately for the calculation of proportions. Shapiro–Wilk tests will be used to assess the normality of data distributions. For normally distributed data, differences between groups will be evaluated using two-tailed *t*-tests. For non-normal distributions, differences between groups will be evaluated using Mann–Whitney *U* tests and Kruskal–Wallis tests. Effect sizes of differences between continuous variables (e.g. TIMI flow, procedure time, etc.) will be expressed using Cohen’s d, which can be interpreted as negligible (d<0.01),very small (0.01≤d<0.2), small (0.2≤d<0.50), medium (0.5≤d<0.80), large (0.8≤d<1.20), very large (1.2≤d<2.0), or huge (d≥2.0). For categorical data, differences between groups will be evaluated using chi-squared tests. All confidence intervals will be presented two-sided with a confidence level of 95%. Denominators used to calculate ratios for the intention-to-treat analysis will be the number of patients allocated to each treatment arm irrespective if they received the allocated treatment. Denominators used in the per-protocol analysis will be the number of patients that received treatment as allocated at enrolment. Denominators used in the as-treated analysis will be the number of patients that received treatment irrespective if the treatment was allocated at enrolment. Differences in success rate (successful wire crossing in ≤60 min with TIMI flow 3 is restored and residual stenosis is <30%) will be expressed in terms of absolute risk (as well as relative risk) to determine if procedures by CCTA CTO-PCA are 20% more successful compared to procedures by conventional Angiography CTO-PCI. Likewise, differences in complications between the two procedures will be expressed using absolute (as well as relative risk). A *p* value <0.05 will be deemed statistically significant. All statistical analyses will be performed using the latest version of R (R Foundation for Statistical Computing, Vienna, Austria).

### Interim analyses {21b}

Not applicable. No interim analyses are planned.

### Methods for additional analyses (e.g. subgroup analyses) {20b}

The analysis of the subgroups of this study will be based on the J-CTO score level, i.e. J-CTO = 2 versus J-CTO ≥3 [7].

### Methods in analysis to handle protocol non-adherence and any statistical methods to handle missing data {20c}

There is no analysis method for non-adherence to the protocol, as the risk for non-adherence is negligible.

### Plans to give access to the full protocol, participant-level data and statistical code {31c}

The research documents concerned by the biomedical research regulations have to be archived by all parties for a 15-year period after completion of the study, in accordance with the Good Clinical Practice guidelines. This archiving will have at least:

1. The protocol and the potential amendments,
2. All eCRFs,
3. All signed informed consent forms with the list or inclusion register in correspondence,
4. The letters of correspondence with the trial steering committee,
5. All specific appendix,
6. The final report of the study from the statistical analysis and the study quality control,
7. The database used for the statistical analysis, and
8. The audit certificates done during the research.

## Oversight and monitoring

### Composition of the coordinating centre and trial steering committee {5d}

The research will be regulated by the standard operating procedures of ELSAN Comité Orientation Scientifique under the supervision of the main author (Dr E La Scala). The trial steering committee will consist of the main author (Dr E La Scala) and the Director of Scientific Research ELSAN (Dr S Klouche).

### Composition of the data monitoring committee, its role and reporting structure {21a}

The data monitoring committee will be composed of at least one person from the clinical research organisation (Ecten) who will be independent from the sponsor and will have no competing interests. The role of the data monitoring committee will be to produce periodic reports to the ELSAN Comité Orientation Scientifique on the progress of the trial.

### Adverse event reporting and harms {22}

All adverse events have to be registered in the eCRF. An adverse event is considered as serious if there is death or life-threatening circumstances, inpatient or prolonged hospitalisation, persistent or significant incapacity or substantial disruption of the ability to conduct normal life functions, congenital anomaly or an event judged as serious by the Investigator.

The medical judgement of the investigator could manage to declare some other events considered as potentially serious (especially some biological anomalies). Expected serious adverse events and unexpected serious adverse events will have to be distinguished.

The investigator must, for each serious or non-serious adverse event, describe them on a form in the eCRF. The etiological diagnostic (rather than symptom), the date of the beginning, the seriousness, the intensity, the action taken with the experimental product or the study procedure, the corrective treatment administered and the causal link between treatment or procedure and serious adverse event have to be described. When the event is considered as serious, the investigator has to inform the trial steering committee immediately after having known of the events. Within 8 days after the declaration, the investigator may provide additional relevant information to the trial steering committee. If a patient declares a serious adverse event, he will be followed until the resolution of this adverse event, a stabilisation considered as tolerable or a return to the previous statement even if the patient is withdrawn from the study. Any serious adverse event associated to a study participant has to be declared from the date of consent form signature and during all the follow-up duration. If the serious adverse event is related to the study procedure, there is no time limitation of participant follow-up.

Risks related with the CT scan, object of our evaluation: as CT scan can be performed as part of standard of care for pathology diagnosis, there is no additional risk related to this investigation performed in the study. Risks will be the same risks as those related to the angiography necessary to visualise coronary arteries before and during the recanalisation procedure, as the contrast medium used is the same:

1. Irradiation
2. Contrast-induced nephropathy in patients with renal insufficiency
3. Allergic reaction to radiographic contrast media

What is the frequency?

1. Allergic reactions to iodinated contrast media are very infrequent: 1.5 events per 1000, of which 2.6% is serious [10].
2. Incidence of renal insufficiency requiring dialysis is < 1% and the development of the complication is correlated with a dose of contrast > 100 ml, clearance of creatinine < 47 ml/min, diabetes [9].

How those risks can be managed or reduced?

1. Reduction of cardiac rate to obtain a good quality exam with low irradiation.
2. Anti-allergic treatment before contrast injection for the atopic patients.

Hydration for patients with chronic renal insufficiency (creatinine clearance <47ml/min), metformin-free period for diabetic patients 24h before and 48h after the contrast injection compared to the volume of contrast medium used during standard angiography and PCI, the volume of contrast medium for the CT scan is low (55ml).

### Frequency and plans for auditing trial conduct {23}

The clinical research associates (CRAs; Ecten, France) mandated by the sponsor, will conduct visits to the investigating centre as planned in the protocol on the patient’s follow-up scheme, from the inclusion of subjects in the centre and with the risk level that has been assigned to the research. The CRA will be responsible, under the Coordinating Investigator, for:

1. Logistics and monitoring of the research
2. Providing reports on its progress and sharing them with the research stakeholders (sponsor, Methodology and Management Centre, etc.)
3. Verifying the completeness of the case report forms (request for additional information, corrections, etc.)

### Plans for communicating important protocol amendments to relevant parties (e.g. trial participants, ethical committees) {25}

Any substantial modification, that is to say, any modification having a significant impact on people protection, on validity conditions and on research results, on the quality and the safety of research treatments, on the scientific documents interpretation used in the justification of the research or on conduct modalities of the research, must cause an amendment proposed to the trial steering committee. Before the setting of the amendment, a favourable recommendation from the CPP is mandatory. Every protocol amendment has to be shared with all investigators included in the research. The investigators commit to respect the amendments. Also, any amendment changing the patients’ care, or the benefits, risks and obligations of the research must cause a new information to note, and a new informed consent form collected with the same way as the initial one.

The trial steering committee can decide to stop the study when he wants for the following reasons:

1. Disability for the investigator to include patients according to the planned schedule.
2. Lack of signed consent form.
3. Missing or wrong data

The trial steering committee has to do an end of study declaration within 90 days after the end of the research when the study is finished. If the clinical trial is early over, this end of study has to be declared to the ANSM within 15 days with a justification about reasons to stop. In case of adverse events judged too serious by the investigator, and could be dangerous for the patients’ health, the investigator can stop the study in accordance with the trial steering committee.

### Dissemination plans {31a}

At the end of the research, each patient included in the study can be informed of the overall results of the research, in compliance with the law, and according to modalities specified in the informed document L1122-1. The main author is the owner of data, analyses and results related to the study, so, none use or relaying to someone cannot be done without his prior agreement.

Results of the study will be submitted for consideration to be published in a PubMed-indexed journal.

## Discussion

This randomised trial will provide insight into whether pre-procedural CCTA as opposed to conventional angiography for planning of CTO-PCI yield higher success rates of wire crossing in ≤60 min. Potential benefits of CCTA include shorter successful procedure times of CTO-PCI leading to less irradiation and contrast medium with lower complication rates.

This will be the second randomised controlled trial to investigate the impact of pre-procedural CCTA on CTO-PCI. Hong et al. [11] conducted a trial to test whether success rates of CTO-PCI increased when including pre-procedural CCTA as part of the treatment regimen, and found that including CCTA increased success rate (187(94%) versus 168(84%)), reduced MACEs (8(4%) vs. 10(5%)), but had higher cardiac-related deaths (1(0.5%) versus 0(0%)) and non-related cardiac deaths (2(1%) versus 0(0%)). Their study design did have some important flaws, which will be addressed in the present study. First, the previous trial enrolled any patient with CTO, whereas the present trial will only enrol patients with complex CTO (J-CTO≥2). The benefits of CCTA in addition to conventional angiography might be realised in more difficult cases of CTO. Moreover, the success of PCI in the previous study was less strict as suboptimal TIMI 2 flow was considered successful, whereas TIMI 3 flow will be considered successful in the present study. Second, the previous study, which was conducted at 12 centres, had a long inclusion period spanning 5 years and 9 months, whereas the present multi-centre study will have a 24-month inclusion period to reduce heterogeneity among patients, and with a higher inclusion rate to avoid potential selection bias. Third, the previous study relied on previous-generation CT scanning systems and protocols, whereas the present study will utilise current-generation equipment and protocols. Finally, the definition of the primary endpoint in the previous study compared success rates for any procedural time, whereas the present study will compare success rates only in cases where procedural time was <60 min.

It is important to note that a CTO-PCI lasting longer than 60 min is not considered a failure if no complications are encountered and TIMI flow 3 with stenosis <30% is achieved. These longer procedures do however present some logistical challenges, which include the need for two catheter laboratories to treat additional acute patients arriving with a myocardial infarction. Longer procedures increase patient irradiation and also require general anaesthesia to reduce patient discomfort, while increasing risks of complications, such as catheter thrombosis or wire perforations. Procedure costs will increase due to the need of more materials.

In conclusion, this trial should help assess whether CCTA is beneficial in planning CTO-PCI for patients with difficult CTO (J-CTO ≥2). This study is the second randomised controlled trial focusing on CCTA, but the first to only focus on treatment of difficult CTO.

## Trial status

Started on 1^st^ of May 2022; NCT04549896; 21/12/2021.

## Data Availability

The investigator has to get the authorisation of all persons implicated in the study research in order to be sure that source data, source documents, research sites, and study reports are directly accessible with the aim of quality control and audit by the trial steering committee. The investigators will make available the documents and individual data strictly necessary for monitoring, quality control and the audit of biomedical research, available to people with access to these documents in accordance with the legal and regulatory provisions in force (Articles L.1121-3 and R5121.13 of the Public Health Code). The trial data will not be made available on a public accessible repository.

## Abbreviations

BMI: Body mass index
CCS: Canadian Cardiovascular Society
CCTA: Coronary computed tomography angiography
CNIL: Commission Nationale Informatique et Liberté
COVID-19: Coronavirus disease
CRA: Clinical research associate
eCRF: Electronic case report form
CT: Computed tomography
CTDI: Computed tomography dose index
CTO: Chronic total occlusion
CT RECTOR: Computed Tomography Registry of Chronic Total Occlusion Revascularization
DAP: Dose area product
DLP: Dose length product
ECG: Electrocardiogram
FAS: Full analysis set
Fr: French
ICH GCP: International Conference on Harmonisation Good Clinical Practice
J-CTO: Japanese Multicentre CTO Registry
MACE: Major adverse cardiac events
MRI: Magnetic resonance image
NYHA: New York Heart Association
PCI: Percutaneous coronary intervention
PPS: Per protocol set
PT: Preferred term
SOC: System organ class
SS: Safety set
TIMI: Thrombolysis in myocardial infarction
